# Qualitative assessment of mobility in older adults: a scoping review of process-oriented behavioural criteria

**DOI:** 10.1186/s11556-026-00408-y

**Published:** 2026-04-09

**Authors:** Natalie Lander, Costas Glavas, Anoohya Gandham, Ana Maria Contardo Ayala, L. Eduardo Cofré Lizama, Yuxin Zhang, Jackson Fyfe, Tao Zhou, David Scott, Robin M. Daly

**Affiliations:** 1 Institute for Physical Activity and Nutrition, School of Exercise and Nutrition Sciences, Deakin University, Melbourne, Australia; 2 Department of Allied Health, School of Health Sciences, Swinburne University of Technology, Hawthorn, Melbourne, Victoria Australia; 3 Department of Medicine (Royal Melbourne Hospital), University of Melbourne, Melbourne, Australia; 4 School of Clinical Sciences at Monash Health, Monash University, Melbourne, Australia

**Keywords:** Process-oriented criteria, Older adults, Qualitative assessment, Functional mobility

## Abstract

**Background/Objectives:**

Mobility decline is a strong predictor of falls, fractures, and disability in older adults and is associated with sarcopenia, kinesiophobia, reduced physical activity, and poorer quality of life. Early identification is essential to mitigate deterioration. Yet, most clinical assessments emphasise performance outcomes (e.g., time, repetitions) and often overlook early qualitative signs of mobility deterioration. Process-oriented assessments evaluating movement quality may offer greater sensitivity but are seldom used given time, resource, and training constraints. Digital technologies could overcome barriers, but practical evidence-based tools remain limited. This review aimed to identify process-oriented behavioural criteria for qualitatively assessing functional mobility in older adults, establishing a foundation for digital mobility assessments.

**Methods:**

Following Joanna Briggs Institute and PRISMA-ScR guidelines, six databases (MEDLINE Complete, APA PsycInfo, CINAHL, Sport Discus, Embase, Web of Science) were searched from inception to September 2025. Six reviewers independently screened studies and extracted data. Inductive analysis identified behavioural criteria and tool characteristics.

**Results:**

Twenty-four studies met inclusion criteria. Most assessed balance and gait using ordinal scales. Berg Balance Scale and Dynamic Gait Index showed high reliability and validity; Mini-BEST and POMA demonstrated moderate properties. Feasibility was inconsistently reported. Limitations included moderate clinical burden, ceiling/floor effects, reliance on trained raters, equipment requirements, and lack of a standardised framework.

**Conclusions:**

Process-oriented assessments are limited by inconsistent psychometric quality, feasibility constraints, and a lack of standardisation, highlighting a critical gap in qualitative assessment.

**Significance/implications:**

Validated process-oriented criteria may enable scalable digital mobility assessment integration, allowing for earlier detection, tailored interventions, reduced falls risk, and streamlined clinical workflows without extensive training or specialised equipment.

**Supplementary Information:**

The online version contains supplementary material available at 10.1186/s11556-026-00408-y.

## Introduction

The global population is ageing rapidly, with projections indicating that by 2050, individuals aged 60 and over will comprise 22% of the global population, over 2.1 billion people [52]. In Australia, more than half (approximately 52%) of adults aged 65 years and older experience a physical disability or functional limitation [44]. These limitations are largely attributable to age- and disease-related declines in neuromuscular function, including reductions in muscle strength, coordination, mobility, balance, and power [10]. Such impairments are associated with increased risk of chronic diseases, loss of independence, hospitalisation, reduced quality of life, and higher mortality risk [10].

Functional ability is defined as the combination of intrinsic capacity, relevant environmental characteristics, and their interactions that enable an individual to do what they have reason to value [59]. Central to functional ability is intrinsic capacity, which encompasses the full range of an individual’s physical and mental capacities [59]. Mobility is recognised as a critical domain of intrinsic capacity [59]. Mobility, an individual’s ability to move independently and perform essential locomotor and functional tasks required for daily living, is determined by multiple interrelated factors, including muscle strength, balance, coordination, and cardiorespiratory endurance. Mobility encompasses functional components such as gait, postural control, and the ability to transition between positions within varying environmental contexts [36, 55]. A decline in mobility is a strong predictor of disability, falls, fractures, and hospitalisation, and may lead to other conditions such as sarcopenia, cardiovascular disease and kinesiophoboa [5, 59, 61]. Kinesiophobia (fear of movement) develops in almost two thirds of older adults, leading to decreased physical activity, accelerating functional decline and reducing life quality [10, 22, 58]. In older age, mobility is particularly important, consequently, it is emphasised in the World Health Organization’s (WHO) framework for healthy ageing, which prioritises mobility preservation to promote autonomy and overall wellbeing [24, 59].

Early identification of mobility decline is vital for the implementation of timely, personalised interventions that can mitigate further deterioration and prevent related adverse outcomes [17]. Accordingly, early identification of mobility decline is also a critical component of the WHO’s Integrated Care for Older People (ICOPE) implementation framework [59], which positions regular assessment (screening) as essential for maintaining functional ability in older adults. Initial screening enables timely detection of early impairments, allowing for prompt intervention before significant functional loss occurs. When screening indicates elevated risk, the ICOPE framework recommends further in-depth assessment, which provides a more comprehensive evaluation of mobility-related domains, including muscle strength, balance, and gait, to inform targeted care and precision physical activity program planning [59].

Despite the crucial role of mobility assessment in preventive geriatric medicine, commonly used tools may be suboptimal for early detection of impairment and the development of personalised interventions to maintain mobility. Widely used assessments, commonly referred to as performance-based assessments [43], such as the Timed Up and Go (TUG) [33] and the Short Physical Performance Battery (SPPB) [14], primarily quantify performance outcomes (e.g., time, repetitions, distance, ability to complete). However, qualitative changes in mobility (e.g., gait variability, compensatory strategies, postural instability) often precede measurable performance decline, suggesting that movement quality may serve as a more sensitive early marker of functional deterioration [13]. Hence, traditional performance or product-oriented assessment tools may risk overlooking critical qualitative features [26]. As a result, subtle but clinically significant impairments may go undetected, delaying intervention and limiting opportunities for prevention.

Process-oriented or judgement-based assessments [43] (e.g. the Balance Evaluation Systems Test [BESTest] [16] and the Tinetti Performance-Oriented Mobility Assessment [POMA] [46], evaluate *how* the movement is performed, or the movement quality, as such, offer a more nuanced understanding of functional capacity. In the context of older adults, these assessments focus on the movement process such as control, coordination, posture, balance, and compensatory strategies during tasks like rising from a chair or walking. This approach has the potential to enable assessors to detect early signs of mobility decline, guide intervention planning, and monitor changes over time with greater sensitivity than outcome-only measures. By identifying specific qualitative movement deficiencies, it can also support precision planning of physical activity programs tailored to individual functional needs, thereby enhancing the relevance and effectiveness of interventions. However, these assessments are underutilised in clinical practice due to their time demands in relation to set up, administration and evaluation, the need for specialist training, and difficulties in standardisation [43]. Although various process-oriented measurement tools have been developed to assess mobility, there is wide variability in the mobility constructs being measured, which limits standardisation and meaningful comparison across studies [4]. Recent advancements in digital health technologies, such as smartphone- or sensor-based mobility assessments are promising [11, 18, 35], However, many existing digital mobility assessments emphasise performance outcomes (e.g., time, distance) rather than movement process or quality, and often rely on relatively simple planar or two-dimensional measures that may not fully capture the complex, dynamic and multidimensional nature of human movement. Further, despite the expanse of assessments available and the technological advancements, many clinical healthcare professionals report that a major barrier to effective care is the lack of appropriate, easy-to-use tools for assessment [8], highlighting a critical gap that limits the integration of mobility assessment into routine clinical practice for the timely identification of mobility decline.

Recent advances in AI-driven, video-based assessment tools use machine-learning algorithms to extract spatiotemporal data from video footage, enabling rapid and automated detection of subtle, qualitative changes in movement patterns that may precede overt mobility impairments. These approaches have been developed and validated in paediatric populations [21]; however, their potential for scalable application and clinical impact in older adults remains unexplored. Realising this potential requires the development of standardised, process-oriented criteria to assess the quality of key mobility tasks in older populations.

To address this gap, we conducted a scoping review to systematically map the literature on process-oriented mobility assessment tools in older adults across community, clinical, home-based and aged-care settings. A scoping review is appropriate for this objective, as it characterises the breadth and depth of the evidence, identifies knowledge gaps, clarifies key concepts, and guides future research and prototype development [32, 50, 51]. This review aims to map the literature using process-oriented mobility assessment tools and identify behavioural criteria that could be used to qualitatively assess functional movements in older adults. By identifying and synthesising existing criteria for qualitative assessment of functional movement in older adults, it lays the foundation for the development of machine learning models capable of automatically detecting early mobility decline. Moreover, the findings may contribute to the standardisation of mobility assessment practices and support the integration of digital health technologies into clinical workflows, ultimately improving outcomes for ageing populations.

## Methods

### Study design

This scoping review was conducted following the Joanna Briggs Institute (JBI) methodology [32] and the Preferred Reporting Items for Systematic Review and Meta-Analyses extension for Scoping Reviews (PRISMA-ScR) [50]. This scoping review did not follow a pre-existing review protocol nor was it registered with PROSPERO. Furthermore, the methodological quality and risk of bias of the included articles were not assessed, as such appraisal is not required for a scoping review.

### Eligibility criteria

This scoping review was guided by the Population, Concept, and Context (PCC) framework as outlined in Joanna Briggs Institute methodology [32]. The PCC framework was used to define both the scope of the review and the eligibility criteria. The population of interest comprised older adults (aged ≥ 65). The concept underpinning this review was *process-oriented behavioural criteria*, defined as the qualitative evaluation of how functional movements are performed rather than the outcome of the movement (e.g. time or repetitions). This includes observable characteristics of movement quality such as postural control, coordination, balance, movement efficiency, and joint and limb control, which reflect underlying motor competence and neuromuscular function. The context included any setting in which mobility or functional movement assessments were conducted, including clinical, community, research, and aged care environments. Eligibility criteria were developed a priori based on this PCC framework and are outlined below.

### Population

Studies involving community-dwelling and institutionalised older adults aged ≥ 65 years were included. This encompassed both healthy individuals and those with acute or chronic conditions (e.g. vestibular disorders, stroke, spinal cord injury, Parkinson’s disease, osteoarthritis, dementia, brain injury, sarcopenia, and history of falls).

### Concept

Studies were included if they described, developed, or evaluated qualitative, process-oriented, or judgement-based behavioural criteria applied to functional movement or mobility assessments. These criteria related to observable aspects of movement performance, including postural control (e.g. trunk alignment, need for support), coordination (e.g. sequencing, symmetry, smoothness), balance and weight transfer, movement efficiency, compensatory strategies (e.g. use of upper limbs or momentum), and joint and limb control (e.g. range of motion, movement trajectory, instability). Studies were excluded if they relied solely on quantitative, outcome-based measures (e.g. time, distance, or total score) without assessment of movement quality.

### Context

Studies conducted in any setting were included, including community (e.g. home-based or public health programs), clinical (e.g. hospitals, outpatient clinics, rehabilitation centres), research (e.g. interventional trials), and aged care settings (e.g. residential care facilities).

### Types of sources

The following peer-reviewed study designs were considered for this review: analytical and descriptive observational studies, prospective and retrospective studies, cross-sectional studies, experimental and quasi-experimental studies and randomised controlled trials. Any published work involving conference proceedings, symposiums, commentaries, editorials or protocols were excluded. Case studies, case reports, case series, qualitative articles, opinion articles or reviews were excluded as our objective was to map tools that specify reproducible, process-oriented mobility tools and report on their characteristics and properties. These designs rarely provide standardised operational definitions, comparative data or adequate sample sizes to evaluate reliability, validity or responsiveness; thus, they could not support the synthesis required for this review.

### Search strategy

The search strategy was designed to identify peer-reviewed, published articles. An initial search of the literature was conducted on MEDLINE Complete and Embase to identify relevant content and published work in the field. Text words contained in both the titles and abstracts of relevant articles were indexed and used to develop and refine the search strategy. Subject headings were identified in relevant articles and were used to further refine the search strategy. A research librarian was consulted in the development of search strategy and translation of the searches across databases. The finalised search strategy, including all indexed keywords and subject headings was adapted for each included database (A1-A3).

Multiple databases were searched for this scoping review, including MEDLINE Complete (via EBSCO Host), APA PsycInfo (via EBSCO Host), CINAHL (via EBSCO Host), SPORT Discus (via EBSCO Host), Embase and Web of Science. The searches were conducted in June 2025, and for the purposes of feasibility, we utilised limiters to only include articles available in English and conducted on humans aged ≥ 65 years.

### Study selection

Following the completion of the searches over the six databases, the identified articles were collected and collated onto EndNote 21 (Clarivate Analytics, Pennsylvania, U.S.), and duplicates were removed. Details of the remaining articles were uploaded onto Covidence (Veritas Health Innovation, Melbourne, Australia) as.RIS files and any remaining duplicates were removed via Covidence.

Once files were imported for screening a total of six independent reviewers (AC, AG, CG, JF, LECL and NL) were involved in screening the titles and abstracts of the articles and assessing them against the inclusion criteria. Two independent reviewers were required to provide one vote on whether to include or exclude a single article. Once articles were screened a moderated consensus was agreed upon between NL and CG to resolve any conflicts in the screening phase and if a consensus could not be reached then a third reviewer, AG, arbitrated the conflicts to meet a consensus.

Full texts of the articles that had passed screening were again reviewed by six independent reviewers (AC, AG, CG, JF, LECL and NL) in detail and assessed against the inclusion criteria. Similarly to screening, two independent reviewers were required to provide one vote on whether to include or exclude a single article. Once full text articles were screened a moderated consensus was agreed upon between CG and NL to resolve any conflicts in the screening phase and if a consensus could not be reached then a third reviewer, AG, arbitrated the conflicts.

Studies were excluded if they: (1) did not include older adults aged ≥ 65 years; (2) did not utilise or demonstrate judgement based, process-oriented criteria for functional movements; (3) did not include outcomes demonstrating motor competency, functional independence, functional movement and mobility, or independent living movement; (4) did not assess or demonstrate judgement-based outcomes with some aspect of qualitative/subjective measure (e.g. motor competency, motor skills, motor performance); (5) did not meet the study design or characteristics criteria.

### Data extraction

Following full-text screening, data were extracted from the included articles by six independent reviewers (AC, AG, CG, JF, LECL and NL), with each article assigned to one primary extractor and one independent checker. The data extraction tool was developed by two reviewers CG and NL and included relevant items pertaining to study characteristics, descriptive characteristics, concept, context and items that addressed the research questions. All relevant items were added to a template form provided by JBI (A4) [32]. Data extracted from all articles included the study title, first author name, year of publication, the journal in which the study was published, study sample sizes and participant characteristics. Assessment details were also recorded, including tools used, types of movement tasks assessed, data collection modalities and descriptions of observations or interventions. Finally, specific information was extracted on process-oriented mobility tools, covering criteria, scoring approaches, rater requirements, psychometric properties, feasibility and acceptability, strength, limitations and stated purposes of tools.

### Data analysis and presentation

We conducted an inductive content analysis to identify, classify and synthesize the key characteristics of the included articles. Behavioural aspects and functional movement demonstrated in the included articles were categorised to better understand each of their components, differences and similarities. Additionally, study descriptions were analysed to identify data collection methodologies, modalities of behavioural and functional assessments including the ‘Tinetti Performance Oriented Mobility Assessment’ (POMA), the ‘miniBESTest: Balance Evaluation Systems Test’ (Mini-BEST), the ‘Functional Movement Screen’ (FMS), the ‘Functional Reach Test’ (FRT), the ‘Dynamic Gait Index’ (DGI), the ‘Functional Gait Assessment’ (FGA), the ‘Berg Balance Scale’ (BBS), the ‘modified Clinical Test for Sensory Interaction on Balance’ (mCTSIB), the ‘Occupational Therapy Functional Assessment Compilation Tool’ (OTFACT) ‘Posturography’ and ‘NeuroCom’ systems.

The results of this scoping review present a description of the included articles and the characteristics of the observations or interventions with a focus on the process-oriented behavioural criteria utilised to qualitatively or subjectively assess functional movements.

## Results

### Article inclusion

**Fig. 1:**
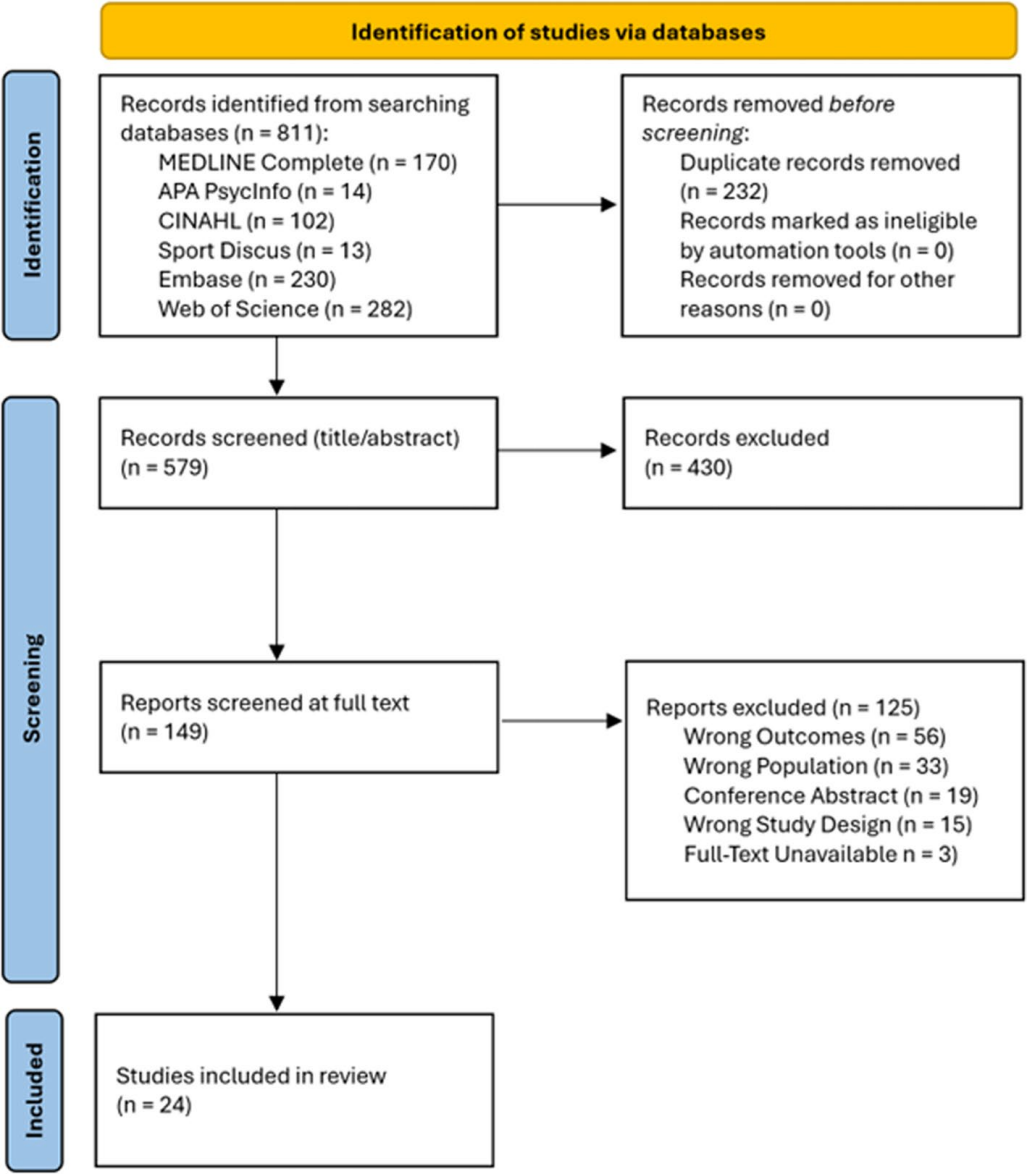
..

A total of 811 records were identified through a search of the six included databases. The PRISMA-ScR flowchart is depicted in Fig. 1, which illustrates the search at different phases of data collection, title/abstract and full text screening. Following the removal of duplicates and completion of title and abstract screening, 149 records were identified for full text review. Throughout full text review, the reasons for exclusions were incorrect outcomes (*n* = 56); populations aged younger than 65 years (n = 32); conference abstracts (*n* = 19); records containing study designs that were not consistent with the inclusion criteria (*n* = 15); and full text of records was not available (*n* = 3). After the full text screening, a total of 24 records were included in this review [1–3, 6, 9, 15, 19, 20, 27, 29–31, 38–40, 42, 45, 47–49, 53, 54, 56, 57].

### Article characteristics

Characteristics of the included articles are illustrated in Table 1. A diverse array of study designs was used, including nine [2, 6, 31, 38, 39, 42, 45, 53, 54] randomised controlled trials, five [1, 3, 9, 15, 49] cross-sectional studies, four [19, 29, 30, 40] prospective studies, three [20, 27, 47] experimental studies, two [56, 57] retrospective study and one [48] quasi-experimental study. The studies were geographically diverse, with seven [6, 27, 30, 47, 48, 56, 57] from the United States of America, three [19, 31, 40] from Germany, two [1, 3] from Brazil, two [45, 53] from India, and one each from England [2], New Zealand [39], Finland [42], Turkey [9], Spain [38], Israel [49], Iran [15], China [54], Japan [29], and Italy [20]. The studies were conducted in diverse settings, including home-based (n = 4; 16.67%), community-based (n = 5; 20.83%), aged care (n = 3; 12.50%) and clinical settings (n = 12; 50%), with sample sizes ranging from 9 to 11,251 participants with or without co-morbidities and a mean age ranging from 65 to 85 years.

**Table 1:** Article characteristics of included studies

Author (year)	Design	Country	Setting	Co-morbidities	Sample Size	Mean (SD) age (years)	Number of women (%)
Ashburn (2019) [2]	RCT	England	Community-based	Parkinson’s disease & falls	474	72 (8)	208 (44%)
Beling (2009) [6]	RCT	USA	Community-based	None	19	80 (6)	8 (42%)
Pavlou (2024) [31]	RCT	Germany	Aged care	Fall-related morbidity	120	73 (3)	70 (60%)
Roig-Casasús (2018) [38]	RCT	Spain	Clinical	Total knee arthroplasty	37	73 (4)	25 (68%)
Rosie (2007) [39]	RCT	New Zealand	Clinical	Multimorbidity	68	85 (4)	47 (71%)
Sihvone (2004) [42]	RCT	Finland	Aged care	None	27	81 (6)	27 (100%)
Sunny (2017) [45]	RCT	India	Aged care	None	40	68 (3)	27 (68%)
Valiv (2025) [53]	RCT	India	Clinical	None	84	69 (3)	N/A*
Wang (2016) [54]	RCT	China	Community-based	None	90	65 (5)	N/A*
La Porta (2022) [20]	Experimental	Italy	Clinical	Parkinson’s disease & stroke	403	76 (6)	264 (66%)
Millage (2017) [27]	Experimental	USA	Clinical	Parkinson’s disease	9	71 (10)	5 (56%)
Tinetti (1997) [47]	Experimental	USA	Home-based	Hip fracture	148	81 (7)	123 (83%)
Tisher (2019) [48]	Quasi-experimental	USA	Home-based	None	38	70 (6)	8 (73%)
Knobe (2016) [19]	Prospective	Germany	Clinical	None	24**	80 (7)	17 (71%)
Nikaido (2019) [29]	Prospective	Japan	Clinical	Idiopathic normal pressure hydrocephalus	68	78 (6)	23 (34%)
Panzer (2011) [30]	Prospective	USA	Community-based	Fall-related morbidity	74	78 (7)	N/A*
Schlenstedt (2016) [40]	Prospective	Germany	Clinical	Parkinson’s disease	66	67 (10)	21 (32%)
Whitney (2013) [56]	Retrospective	USA	Home-based	None	10,953	84 (7)	6432 (59%)
Whitney (2013) [57]	Retrospective	USA	Home-based	Multimorbidity	11,251	84 (7)	6751 (69%)
Almeida (2014) [1]	Cross-sectional	Brazil	Clinical	Parkinson’s disease	171	72 (7)	89 (52%)
Barbieri (2012) [3]	Cross-sectional	Brazil	Clinical	Parkinson’s disease	54	66 (9)	25 (46%)
Demirbüken (2012) [9]	Cross-sectional	Turkey	Clinical	T2DM & neurological dysfunction	59	81 (7)	N/A*
Hayati (2025) [15]	Cross-sectional	Iran	Community-based	Frailty	161	68 (7)	70 (44%)
Toledano-Shubi (2025) [49]	Cross-sectional	Israel	Clinical	Cardio-respiratory disease & T2DM	48	73 (5)	33 (69%)

^*^ Study did not report sex-stratification

^**^Data from the treatment group only was extracted, as the control group consisted of young healthy adults

### Characteristics of process-oriented mobility tools

**Fig. 2:**
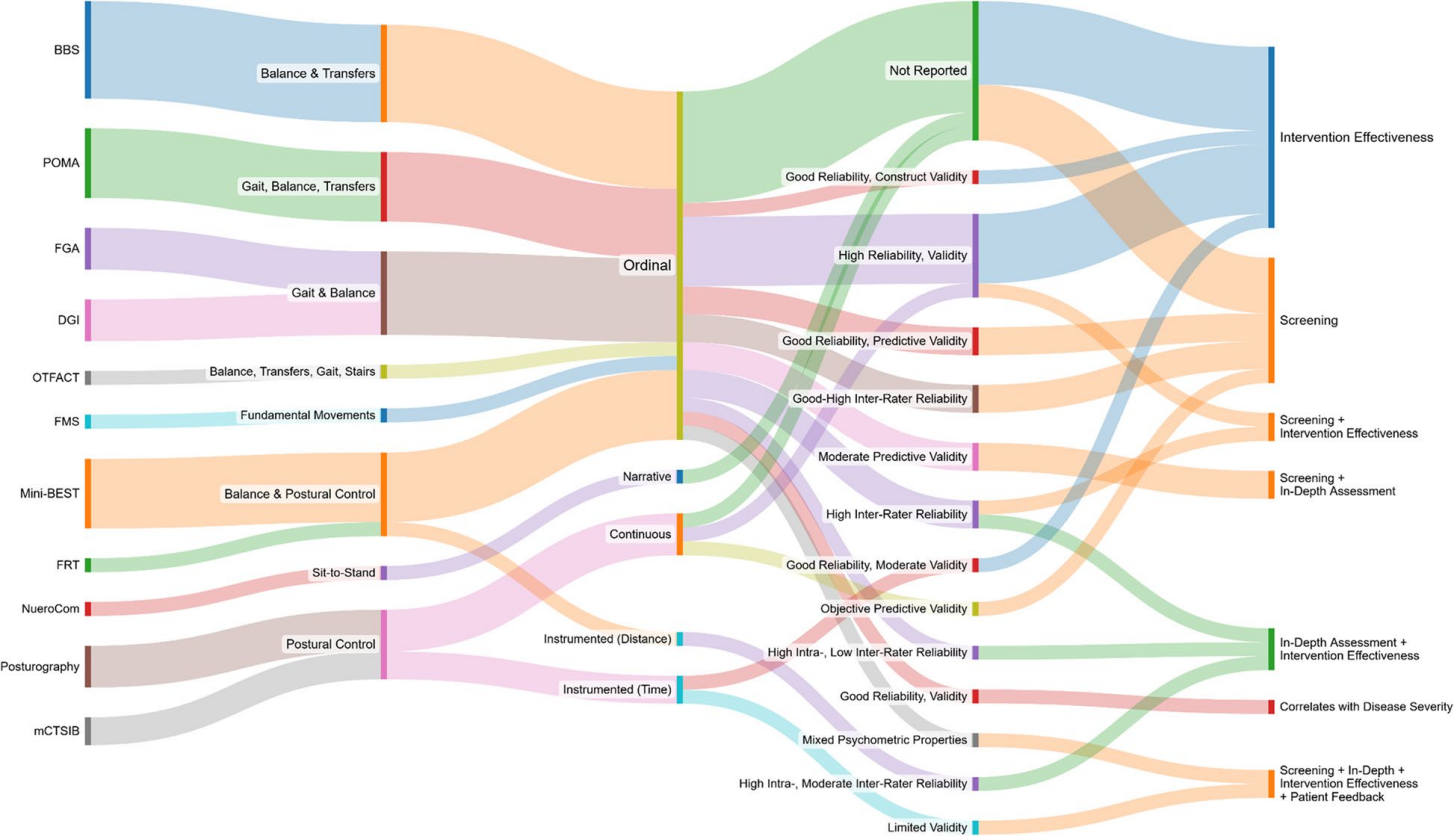
..

The components and characteristics of the process-oriented mobility tools used in the included studies are outlined in Table 2 and Fig. 2. A wide range of process-oriented tools were used, including the Mini-BEST [2, 20, 40, 49, 53], BBS [3, 38, 39, 42, 48, 49, 56, 57], POMA [15, 19, 20, 30, 56, 57], DGI [1, 45, 56, 57], FGA [27, 29, 31], FRT [53], FMS [54], OTFACT [47] and mCTSIB [48, 56, 57]. Technology-based process-oriented assessments, including Posturography [6, 30, 42] and the NeuroCom system [9], were also used.

**Table 2:** Specific characteristics and properties of process-oriented mobility tools as applied within individual studies

Author (year)	Tool	Movement	Scoring Approach	Rater Requirements	Psychometric Properties
Ashburn (2019) [2]	Mini-BEST	Balance & Postural Control	Ordinal Scale	Not reported	Not reported
Beling (2009) [6]	Posturography	Balance & Postural Control	Continuous instrumented measurement	Not reported	Not reported
Pavlou (2024) [31]	FGA	Gait & Balance	Ordinal Scale	Raters were trained on the use of the FGA	Good reliability, construct and criterion validity and sensitivity
Roig-Casasús (2018) [38]	BBS	Balance & Transfers	Ordinal Scale	Raters were trained on the use of the BBS	High intra- and inter-rater reliability, concurrent validity, predictive validity, and construct validity
Rosie (2007) [39]	BBS	Balance & Transfers	Ordinal Scale	Raters were trained on the use of the BBS	High intra- and inter-rater reliability, predictive and construct validity
Sihvone (2004) [42]	Posturography & BBS	Balance & Postural Control	Posturography: Continuous instrumented measurement BBS: Ordinal Scale	Raters were trained on the use of the Posturography software and the BBS	High reliability, concurrent and predictive validity for the BBS, note reported for Posturography
Sunny (2017) [45]	DGI	Gait & Balance	Ordinal Scale	Raters were trained on the use of the DGI	High reliability, validity, and responsiveness
Valiv (2025) [53]	Mini-BEST & FRT	Balance & Postural Control	Mini-BEST: Ordinal Scale FRT: Continuous distance measurement	Not reported	High intra-validity for both assessments, low inter-validity for Mini-BEST and moderate for FRT
Wang (2016) [54]	FMS	Squat, Hurdle Step, Inline Lung, Shoulder Mobility, Straight Leg Raise, Trunk Stability Push Up, Rotary Stability	Ordinal Scale	Raters were trained on the use of FMS	High inter-rater reliability
La Porta (2022) [20]	Mini-BEST & POMA	Gait, Balance, Postural Control, Transfers, Sit-to-Stand	Mini-BEST: Ordinal Scale POMA: Ordinal Scale	Raters were trained physical therapists	Not reported
Millage (2017) [27]	FGA	Gait & Balance	Ordinal Scale	Raters were trained physical therapists	Not reported
Tinetti (1997) [47]	OTFACT	Balance, Transfers, Gait, Stair Climb	Ordinal Scale	Raters were trained physical therapists	High inter-rater reliability
Tisher (2019) [48]	BBS & mCTSIB	Balance, Transfers, Postural Control	BBS: Ordinal Scale mCTSIB: Continuous time-based duration scoring	Raters were trained on the use of the BBS and mCTSIB	High reliability and validity for BBS, good reliability and moderate validity for mCTSIB
Knobe (2016) [19]	POMA	Gait, Balance, Postural Control, Transfers, Sit-to-Stand	Ordinal Scale	Not reported	Good concurrent validity, moderate predictive validity, high construct validity
Nikaido (2019) [29]	FGA	Gait, Balance, Postural Control	Ordinal Scale	Raters were trained physical therapists	Not reported
Panzer (2011) [30]	POMA & Posturography	Gait, Balance, Postural Control, Transfers, Sit-to-Stand	POMA: Ordinal Scale Posturography: Continuous instrumented measurement	Not reported	Good reliability, and predictive validity for POMA and Posturography
Schlenstedt (2016) [40]	Mini-BEST	Balance & Postural Control	Ordinal Scale	Raters were trained on the use of the Mini-BEST and had prior experience	Moderate predictive validity
Whitney (2013) [56]	POMA & DGI	Gait, Balance, Postural Control, Transfers, Sit-to-Stand	POMA: Ordinal Scale DGI: Ordinal Scale	Raters were physical therapists and trained in the use of both POMA and DGI	Not reported
Whitney (2013) [57]	BBS, POMA, DGI, mCTSIB	Gait, Postural Control, Transfers, Sit-to-Stand	BBS: Ordinal Scale POMA: Ordinal Scale DGI: Ordinal Scale mCTSIB: Continuous time-based duration scoring	Raters were physical therapists and were trained in the use of the BBS, POMA, DGI and mCTSIB	Mixed results for inter-rater reliability, high construct validity, limited concurrent and predictive validity
Almeida (2014) [1]	DGI	Gait & Balance	Ordinal Scale	Not reported	Not reported
Barbieri (2012) [3]	BBS	Balance & Transfers	Ordinal Scale	Raters were trained on the use of the BBS	Good validity and reliability
Demirbüken (2012) [9]	NeuroCom	Sit-to-Stand	Narrative Description	Raters were trained in NeuroCom software analysis	Not reported
Hayati (2025) [15]	POMA	Gait, Balance, Postural Control, Transfers, Sit-to-Stand	Ordinal Scale	Not reported	Good reliability and predictive validity
Toledano-Shubi (2025) [49]	Mini-BEST, BBS	Balance, Transfers, Postural Control	Mini-BEST: Ordinal Scale BBS: Ordinal Scale	Raters were experience physiotherapists	Good to high inter-rater reliability

*BBS* Berg Balance Scale, *DGI* Dynamic Gait Index, *FGA* Functional Gait Assessment, *FMS* Functional Movement Screen, *FRT* Functional Reach Test, *Mini-BEST* Mini Balance Evaluation Systems Test, *mCTSIB* Modified Clinical Test of Sensory Interaction on Balance, *OTFACT* Occupational Therapy Functional Assessment Compilation Tool, *POMA* Performance Oriented Mobility Assessment

Scoring approaches varied between assessments, ranging from ordinal scales used in most clinical tests [1–3, 15, 19, 20, 27, 29–31, 38–40, 42, 45, 47–49, 53, 54, 56, 57] to continuous instrumented measures [6, 30, 42, 48, 53, 56, 57], with one study reporting the use of a narrative description [9]. In most studies, raters were trained in the administration of the respective assessment tools [3, 9, 20, 27, 29, 31, 38–40, 42, 45, 47–49, 54, 56, 57], while some studies did not report rater training requirements [1, 2, 6, 15, 19, 30, 53].

Psychometric properties of the assessments were inconsistently reported and/or varied between assessments (Table 2). Several tools demonstrated high reliability and validity, such as the BBS [38, 39, 42, 48] and the DGI [45], while others indicated good or moderate properties, including the POMA [15, 19, 30] and Mini-BEST [40, 49]. Some studies, particularly those using Posturography [6, 42] or the NeuroCom system [9] did not report test psychometric properties. Mixed results were observed for one study employing combined assessments [56, 57], with strong construct validity but limited concurrent and predictive validity.

Table 3 summarises the process-oriented criteria constituting each mobility assessment tool. Although the specific criteria differed between measures, several recurring domains were evident, most notably postural control, coordination, balance and weight transfer, and the overall efficiency and quality of movement execution [2, 38, 54]. Tools differed in breadth and emphasis of these domains. Broader clinical measures including the Mini-BEST and BBS generally evaluated multiple aspects of movement performance across tasks, such as anticipatory and reactive control, sensory orientation and dynamic stability [2, 38, 40, 48, 49]. By contrast, more task-specific tools such as the DGI, FGA and POMA focused on discrete components of mobility, with greater emphasis on gait adaptability, turning, obstacle negotiation and transitional movements [1, 15, 19, 27, 29, 30, 45]. Instrumented assessments offered a complimentary perspective by quantifying underlying balance-related processes through indices such as centre of pressure displacement, sway characteristics, and sensory integration or reweighting [6, 9, 42, 48]. These approaches provided insight into control mechanisms that may not be readily discernible through observational assessment alone. In addition, several less frequently used tools extended process-oriented assessment to other movement contexts, including functional strength and reach-based tasks, incorporating criteria related to alignment, compensatory movement strategies, and movement control [47, 53, 54].

**Table 3:** Synthesis of process-oriented assessment criteria by tool

Author (year)	Tool	Process-Oriented mobility assessment Criteria
Ashburn (2019) [2]	Mini-BEST	Assesses quality across 4 domains: anticipatory (timely, well-sequenced weight shift), reactive (quick, appropriately scaled compensatory steps), sensory orientation (stable alignment to gravity under altered inputs), and dynamic gait (efficient pivots and obstacle navigation)
Beling (2009) [6]	Posturography	Judges how balance is controlled in static trials, characterises centre of pressure behaviour and strategy selection (ankle vs hip), evaluates sensory selection and vestibular inputs, postural responses and recovery
Pavlou (2024) [31]	FGA	Assesses how gait adapts to task demands across 10 items: initiation and continuity, speed modulation, head and pivot turn, obstacle negotiation, narrow-base walking, eye-closed walking, backward ambulation and stair negotiation
Roig-Casasús (2018) [38]	BBS	Assesses quality across 14 tasks: momentum control and sequencing, steadiness and alignment in standing, controlled reach and limits of stability, efficient turning with minimal steps, and precise placement and stability
Rosie (2007) [39]	BBS	Assesses quality across 14 tasks: momentum control and sequencing, steadiness and alignment in standing, controlled reach and limits of stability, efficient turning with minimal steps, and precise placement and stability
Sihvone (2004) [42]	Posturography & BBS	Posturography: Judges how balance is controlled in static trials, characterises centre of pressure behaviour and strategy selection (ankle vs hip), evaluates sensory selection and vestibular inputs, postural responses and recovery BBS: Assesses quality across 14 tasks: momentum control and sequencing, steadiness and alignment in standing, controlled reach and limits of stability, efficient turning with minimal steps, and precise placement and stability
Sunny (2017) [45]	DGI	Evaluates how gait is executed while adapting to task demands: initiation and continuity, speed scaling, stable head and pivot turns, obstacle negotiation, and stair ascent and decent
Valiv (2025) [53]	Mini-BEST & FRT	Mini-BEST: Assesses quality across 4 domains: anticipatory (timely, well-sequenced weight shift), reactive (quick, appropriately scaled compensatory steps), sensory orientation (stable alignment to gravity under altered inputs), and dynamic gait (efficient pivots and obstacle navigation) FRT: Assesses how forward reach is achieved from a fixed base: controlled anterior centre of mass, arm maintenance at 90 degrees, stable trunk and pelvis without flexion, rotation or shoulder protraction, no stepping, grasping or base-widening, minimal sway and steady return to upright
Wang (2016) [54]	FMS	Assesses performance on seven domains: deep squat, hurdle step, in-line lunge, shoulder mobility, active straight-leg raise, trunk stability push-up, and rotary stability. Records alignment, sequencing, symmetry, range and control, compensations and pain responses
La Porta (2022) [20]	Mini-BEST & POMA	Mini-BEST: Assesses quality across 4 domains: anticipatory (timely, well-sequenced weight shift), reactive (quick, appropriately scaled compensatory steps), sensory orientation (stable alignment to gravity under altered inputs), and dynamic gait (efficient pivots and obstacle navigation) POMA: Assesses quality through balance and gait, controlled sit-to-stand transfers, postural responses, 360-degree turns, initiation of gait, continuity, stride length and height, path control, trunk stability and pace
Millage (2017) [27]	FGA	Assesses how gait adapts to task demands across 10 items: initiation and continuity, speed modulation, head and pivot turn, obstacle negotiation, narrow-base walking, eye-closed walking, backward ambulation and stair negotiation
Tinetti (1997) [47]	OTFACT	Records how tasks are performed through observed performance (no deficit, partial deficit, total deficit), and branches to sub-criteria to localise where the activity breaks down and distinguishes environment-free from environment-adjusted performance
Tisher (2019) [48]	BBS & mCTSIB	BBS: Assesses quality across 14 tasks: momentum control and sequencing, steadiness and alignment in standing, controlled reach and limits of stability, efficient turning with minimal steps, and precise placement and stability mCTSIB: Describes how balance is controlled across four domains: sway amplitude/velocity, strategy selection (ankle vs hip), sensory reweighting when vision or somatosensation is reduced and assistance behaviours (hand-seeking, stepping, base widening)
Knobe (2016) [19]	POMA	Assesses quality through balance and gait, controlled sit-to-stand transfers, postural responses, 360-degree turns, initiation of gait, continuity, stride length and height, path control, trunk stability and pace
Nikaido (2019) [29]	FGA	Assesses how gait adapts to task demands across 10 items: initiation and continuity, speed modulation, head and pivot turn, obstacle negotiation, narrow-base walking, eye-closed walking, backward ambulation and stair negotiation
Panzer (2011) [30]	POMA & Posturography	POMA: Assesses quality through balance and gait, controlled sit-to-stand transfers, postural responses, 360-degree turns, initiation of gait, continuity, stride length and height, path control, trunk stability and pace Posturography: Judges how balance is controlled in static trials, characterises centre of pressure behaviour and strategy selection (ankle vs hip), evaluates sensory selection and vestibular inputs, postural responses and recovery
Schlenstedt (2016) [40]	Mini-BEST	Assesses quality across 4 domains: anticipatory (timely, well-sequenced weight shift), reactive (quick, appropriately scaled compensatory steps), sensory orientation (stable alignment to gravity under altered inputs), and dynamic gait (efficient pivots and obstacle navigation)
Whitney (2013) [56]	POMA & DGI	POMA: Assesses quality through balance and gait, controlled sit-to-stand transfers, postural responses, 360-degree turns, initiation of gait, continuity, stride length and height, path control, trunk stability and pace DGI: Evaluates how gait is executed while adapting to task demands: initiation and continuity, speed scaling, stable head and pivot turns, obstacle negotiation, and stair ascent and decent
Whitney (2013) [57]	BBS, POMA, DGI, mCTSIB	BBS: Assesses quality across 14 tasks: momentum control and sequencing, steadiness and alignment in standing, controlled reach and limits of stability, efficient turning with minimal steps, and precise placement and stability POMA: Assesses quality through balance and gait, controlled sit-to-stand transfers, postural responses, 360-degree turns, initiation of gait, continuity, stride length and height, path control, trunk stability and pace DGI: Evaluates how gait is executed while adapting to task demands: initiation and continuity, speed scaling, stable head and pivot turns, obstacle negotiation, and stair ascent and decent mCTSIB: Describes how balance is controlled across four domains: sway amplitude/velocity, strategy selection (ankle vs hip), sensory reweighting when vision or somatosensation is reduced and assistance behaviours (hand-seeking, stepping, base widening)
Almeida (2014) [1]	DGI	Evaluates how gait is executed while adapting to task demands: initiation and continuity, speed scaling, stable head and pivot turns, obstacle negotiation, and stair ascent and decent
Barbieri (2012) [3]	BBS	Assesses quality across 14 tasks: momentum control and sequencing, steadiness and alignment in standing, controlled reach and limits of stability, efficient turning with minimal steps, and precise placement and stability
Demirbüken (2012) [9]	NeuroCom	Characterises sensory reweighting and strategy selection (ankle vs hip) under sway-referenced conditions, records centre-of-gravity path accuracy, speed and smoothness through biofeedback. Assesses weight-bearing squat/unilateral stance and functional tasks (sit-to-stand, step and turn), noting loading symmetry, response latency, path control and assistance requirements
Hayati (2025) [15]	POMA	Assesses quality through balance and gait, controlled sit-to-stand transfers, postural responses, 360-degree turns, initiation of gait, continuity, stride length and height, path control, trunk stability and pace
Toledano-Shubi (2025) [49]	Mini-BEST, BBS	Mini-BEST: Assesses quality across 4 domains: anticipatory (timely, well-sequenced weight shift), reactive (quick, appropriately scaled compensatory steps), sensory orientation (stable alignment to gravity under altered inputs), and dynamic gait (efficient pivots and obstacle navigation) BBS: Assesses quality across 14 tasks: momentum control and sequencing, steadiness and alignment in standing, controlled reach and limits of stability, efficient turning with minimal steps, and precise placement and stability

*BBS* Berg Balance Scale, *DGI* Dynamic Gait Index, *FGA* Functional Gait Assessment, *FMS* Functional Movement Screen, – Functional Reach Test, *Mini-BEST* Mini Balance Evaluation Systems Test, *mCTSIB* Modified Clinical Test of Sensory Interaction on Balance, *OTFACT* Occupational Therapy Functional Assessment Compilation Tool, *POMA* Performance Oriented Mobility Assessment

Collectively, these findings indicate that, despite variation in content and format, the included tools shared a common emphasis on evaluating the manner in which movement was performed, while differing in the domains prioritised, the level of detail captured and the method of assessment.

### Feasibility, acceptability and purpose of tools

#### Feasibility and acceptability

Components of these process-oriented mobility tools relating to their feasibility and acceptability are outlined in Supplementary Table S2. Reporting was inconsistent across studies, with many omitting explicit measures of feasibility and acceptability [1, 2, 6, 9, 20, 27, 29, 30, 47, 53, 56, 57]. Several tools were reported to be ‘relatively quick’ to administer and acceptable for both clinicians and participants [3, 19, 31, 38–40, 45, 49, 54]. However, administrative times, where reported, ranged from 10 to 20 min per person. The BBS [3, 38, 39, 42, 49] was most consistently reported as feasible, requiring a relatively short time to administer, and documented as widely accepted in clinical practice due to its simplicity, low cost and clear protocol. One study [49] reported the Mini-BEST to be feasible with a short administration time and standardised equipment. Similarly, one study [31] described the FGA as practical, well-accepted and relatively short to administer, however it imposed a moderate burden on clinicians and participants, which likely reflects practical considerations such as clinician training and the need for real-time supervision and manual scoring. The POMA [15, 19, 56, 57] and the DGI [45, 56, 57] were generally regarded as simple, quick and effective tools requiring minimal equipment to administer. Additionally, the FMS [54] was described as relatively easy to administer and adaptable for remote assessments through videoconferencing.

#### Purpose of the tools

Regarding the purpose of the tools used in the selected studies, most assessments were used to either measure intervention effectiveness [2, 6, 20, 27, 31, 38, 39, 42, 45, 47, 48, 53, 54, 56, 57] or for screening older adults [1, 9, 15, 19, 29, 30, 40, 45, 49, 54, 56, 57]. A smaller subset of tools also applied in-depth assessment contexts, providing detailed feedback to personalise interventions [19, 40, 47, 53, 56, 57].

#### Strengths of the tools

Strengths were most frequently noted for tools with established clinical uptake and multidimensional scope. The BBS was widely recognised as clinically adopted, low-cost, and applicable to diverse populations without the need for formal certifications [3, 49]. The Mini-BEST was identified as multidimensional, standardised, and capable of assessing a range of balance domains [40, 49]. POMA and FGA were valued for their practicality in assessing balance, gait and frailty risk [15, 19, 29, 30, 56, 57], while Posturography was highlighted as an objective, assessor-independent measure of postural control (balance) [30, 42].

#### Limitations of the tools

Several limitations were identified across tools. Studies using the BBS indicated ceiling and floor effects, the inability to assess speed, and its lack of disease specificity [3, 49, 56, 57]. Mini-BEST and FGA assessments were reported as potentially challenging for participants with severe mobility impairments or limited adaptability [29, 31, 40, 49]. Posturography was restricted by reliance on lab-based equipment and limited feasibility data [30, 42]. The POMA was reported to have mixed findings of reliability and validity, and challenges with scoring approaches (e.g., subjective scoring, limited sensitivity, and variability across populations) [15, 19, 56, 57].

#### Summary

Across studies, reporting of feasibility and acceptability was inconsistent, with many studies providing limited or no explicit evaluation of these factors. Where reported, most process-oriented mobility tools were described as relatively quick and acceptable to administer, typically requiring 10–20 min, with tools characterised by simplicity, low cost, and minimal equipment demonstrating greater feasibility for routine clinical use. However, feasibility varied depending on practical demands such as clinician involvement, training, space, and scoring requirements, with some tools imposing additional burden due to the need for real-time supervision or manual scoring. In terms of purpose, tools were most commonly used for intervention evaluation and screening, with fewer applied for more detailed, individualised assessment. Strengths were generally associated with tools that were multidimensional, clinically established, and easy to implement, while limitations highlighted trade-offs between practicality and depth of assessment. Common challenges included ceiling and floor effects, limited sensitivity to change, reliance on subjective scoring, and reduced feasibility for individuals with more severe impairments or in settings requiring specialised equipment. Overall, findings indicate a balance between usability and comprehensiveness, with no single tool optimally addressing all domains of movement quality, feasibility, and clinical applicability. The heterogeneity and overlap observed across instruments provided the basis for a subsequent inductive, interpretive synthesis, through which overarching domains of process-oriented mobility assessment were derived, as outlined in the Discussion.

## Discussion

This scoping review demonstrates that a broad range of process-oriented mobility tools have been applied to qualitatively assess functional movement across different settings in older adults, with most targeting domains such as balance, postural control, and gait adaptability. Core instruments, including the Mini-BEST [12], POMA [46], DGI [41], FMS [7], and FGA [60], commonly employed ordinal, task-based scoring to evaluate movement quality. In contrast, technology-based assessments, such as Posturography, the NeuroCom system, and the mCTSIB, provided continuous measurement of movement quality and characteristics, capturing objective measures of sensorimotor integration through the centre-of-pressure behaviour. Taken together, this diversity in measurement approaches suggests the field lacks a unified conceptualisation of movement quality, with tools operationalising mobility through differing theoretical lenses rather than a shared framework.

Despite the breadth of assessments tools identified, several limitations were evident. Psychometric reporting was inconsistent across studies and technology-based instruments frequently lacked evaluation of predictive validity. High reliability and validity are critical, as inconsistent measurement can undermine clinical decision-making and limit the capacity to detect true change over time [28]. Further, feasibility and acceptability were variably described; where reported, tools generally took 10–20 min to administer, required specialised equipment, and relied on trained raters, potentially reducing feasibility, reliability and constraining their broader uptake in clinical practice. Ensuring assessments are easy to use, practical and time-efficient is critical for widespread clinical uptake [8, 23]. Finally, the absence of standardisation across instruments, which often assess only narrow and isolated components of movement, reduces comparability across studies and hinders implementation in practice [37]. These limitations highlight the need for a feasible, reliable, and standardised framework of movement quality that integrates multiple domains of mobility for clinical application. Collectively, these limitations point to a broader translational gap, where the proliferation of tools has not been matched by their readiness for routine clinical integration or scalability across settings.

Traditional assessments predominantly adopt an outcome or product-oriented perspective, quantifying outcomes such as time, distance, or repetitions. While objective and practical, these measures may overlook subtle yet clinically meaningful deficits in coordination, sequencing, and postural alignment that often precede overt performance decline [26]. Reliance on product-oriented metrics risks misclassification, either failing to detect impairment or masking emerging decline, thereby limiting opportunities for targeted, preventative interventions. In contrast, process-oriented tools provide richer insights into how movements are executed and offer more actionable information to guide clinical decisions [26]. These assessments have the potential to inform the tailoring of specific rehabilitation and precision exercise programs, supporting strategies to support physical activity and slow functional mobility decline [26, 59]. However, the findings of this review highlight that most instruments were applied primarily for screening or to measure intervention effectiveness, with only a small subset used for detailed assessment, personalised feedback or precision programming. This narrow application constrains their translational value, limiting the ability to provide individualised feedback, guide tailored interventions, and fully leverage their capacity to identify subtle mobility deficits that may precede quantitatively measured functional decline. This suggests that the current use of process-oriented assessments remains largely descriptive rather than decision-oriented, representing a missed opportunity to embed movement quality metrics within personalised and preventative models of care.

The introduction of digital tools offers promising opportunities to enhance clinical application by providing more detailed and objective evaluations of mobility. In this review, technologies such as Posturography, the NeuroCom system, and the mCTSIB enabled sophisticated assessments of balance control and sensorimotor integration through the centre-of-pressure behaviour, while the OTFACT provided graded profiling of performance deficits. Despite their potential, a major limitation is the lack of reported psychometric properties, constraining interpretability and limiting broader clinical adoption [25, 34]. Additional practical barriers, such as high cost, equipment complexity, and the need for specialist training, further restrict routine integration. Addressing these gaps through validation studies and user-focused implementation strategies is essential to maximise translational potential and align digital tools within a standardised assessment framework [34]. Without concurrent advances in validation and implementation, there is a risk that digital tools will further fragment the field rather than consolidate it, reinforcing the need for frameworks that align technological innovation with clinical utility.

This scoping review identified multiple mobility domains assessed by instruments, including anticipatory and reactive postural control, dynamic gait adaptability, sequencing, and trunk alignment (e.g., Mini-BEST, BBS). Other measures had a narrower focus, such as the POMA on transfers and gait, the DGI and FGA on adaptability tasks, or posturography and mCTSIB on sensory reweighting and sway dynamics. Less frequently applied tools, including OTFACT, NeuroCom, FMS, and FRT, expanded assessment into deficit profiling, biofeedback, fundamental movement quality, and anterior reach mechanics. Synthesising across instruments highlights seven core domains of process-oriented mobility assessment can be delineated. These domains should be interpreted as a higher-order, conceptual synthesis of patterns observed across instruments, rather than as discrete findings directly extracted from the included studies and include: (1) postural control and stability; (2) coordination, timing, and sequencing; (3) gait adaptability and dynamic balance; (4) strategy selection and sensory integration; (5) trunk and segmental control; (6) task-specific functional performance; and (7) cognitive-motor integration, the latter notably underrepresented across tools. While these domains provide a comprehensive conceptual framework, current instruments variably capture only subsets, leaving gaps in comparability, generalisability, and standardisation. This fragmentation limits the capacity to detect early, qualitative changes in mobility that may precede overt decline. Establishing a unified framework of process-oriented mobility criteria from this, therefore, represents a critical next step to enhance measurement precision, support digital and machine learning applications, and strengthen the clinical utility of mobility assessment in ageing populations. Importantly, articulating these domains provides a foundation for shifting the field toward a more integrated and theoretically grounded approach to mobility assessment, with potential to inform both standardised clinical practice and the development of next-generation assessment technologies.

### Implications for research and practice

Our findings have several important implications for practice and research. For clinicians, the absence of a standardised and comprehensive framework for assessing movement quality means that current assessments may provide only partial insight into an individual’s mobility profile. This highlights the need for practitioners to adopt a more multidimensional approach to assessment, where possible, and to interpret results with an awareness of the specific domains captured, and omitted, by each tool. Greater consideration should also be given to the feasibility of tools in routine settings, including time, training, and resource requirements, to support consistent and scalable implementation.

For researchers, these findings emphasise the need to move beyond continued development of isolated tools toward consolidation and validation of core mobility domains. Establishing consensus around key constructs of movement quality would support greater standardisation, improve comparability across studies, and strengthen the translation of evidence into practice. There is a particular need for rigorous psychometric evaluation of process-oriented tools, including predictive validity for clinically meaningful outcomes (e.g., falls) and responsiveness to change over time.

Finally, while emerging technologies such as wearable sensors, video analytics, and artificial intelligence offer significant potential to enhance objectivity and scalability, their impact depends on alignment with clinically meaningful constructs and usability in real-world contexts. Accordingly, both research and practice should prioritise approaches that ensure these technologies are interpretable, feasible, and capable of informing clinical decision-making, supporting their integration into routine assessment and care.

### Future research

Findings from the scoping review may inform the future development of an AI-powered, device-agnostic digital solution that could be designed to unify the seven core domains within a standardised framework. Such a solution could leverage video-based machine learning to extract spatial and temporal data, enabling three-dimension (3D) trajectory mapping and incorporating metrics such as velocity, coordination, and body positioning. This has been successfully achieved in paediatric populations [21], and is able to detect subtle movement impairments that are often missed by conventional assessments, thereby supporting timely and individualised interventions. A similar scalable, web-based architecture could enhance accessibility and cost-effectiveness of mobility assessment in aging populations. By integrating the consolidated domains of process-oriented assessment, as identified in this review, into a cohesive, AI-driven platform, this prospective solution could address current fragmentation in mobility evaluation and contribute to more responsive, preventative models of care, particularly for ageing populations.

### Strengths and limitations

This scoping review adhered to JBI methodology and PRISMA-ScR reporting, utilised a librarian-refined, multi-database search strategy, and mapped a broad PCC framework spanning community, clinical, research and aged-care settings. Screening was rigorous, six independent reviewers conducted screening of titles and abstracts, and full-text with moderated consensus and third-party arbitration where necessary, thereby reducing selection error. Data-extraction was performed based on the JBI extraction tool, tailored to process-oriented criteria and inductive content analysis classified tools and criteria, scoring approaches, rater requirements, psychometric properties, feasibility and acceptability.

The findings of this review should be interpreted with caution, and several limitations must be acknowledged. The review was limited to English-language publications, excluded grey literature and conference proceedings, which may have introduced publication and retrieval biases. The exclusion of qualitative studies, case series and case reports likely omitted nuanced descriptions of process-oriented criteria that may have informed framework development. Considerable heterogeneity in study design, populations, and reporting prevented cohesive synthesis of the included studies. Moreover psychometric, feasibility and acceptability data were inconsistently reported, with technology-based tools often lacking external validation. Evidence for remote assessments was sparse and methodologically constrained, particularly regarding the accurate capture of gait speed and spatiotemporal parameters.

## Conclusions

This scoping review highlights the wide range of process-oriented tools used to assess mobility in older adults but also exposes critical limitations that may constrain their clinical uptake. These include psychometric inconsistencies, feasibility challenges, reliance on trained raters, and the absence of a unified framework. Product-oriented measures, while more commonly applied, fail to capture the nuanced, qualitative changes in movement that often signal early functional decline. To bridge this gap, there is a pressing need to consolidate key movement quality domains into a standardised framework, supported by robust psychometric validation and feasibility data. Such an approach would enhance comparability across tools, improve clinical decision-making, and enable the integration of emerging digital technologies. Establishing a standardised framework for process-oriented assessment therefore represents a crucial step toward more precise, personalised, and preventive models of care in mobility assessment.

## Competing interests

The authors declare no competing interests.

## Supplementary Information

Supplementary Material 1.

Supplementary Material 2.

## Data Availability

No datasets were generated or analysed during the current study.

## Appendix

**Table 4:** Search strategy for EBSCO Host databases, including MEDLINE complete, APA PsycInfo, CINAHL and sport discus

Search No.	Concept	Search Terms	MEDLINE (n)*	PsycInfo (n)*	CINAHL (n)*	SPORT Discus (n)*
1	Population	(“older adult*” OR elderly OR elder OR “old age*” OR “65 years old”)	492,502	146.662	210,568	28,669
2	Population	(MH "Aged+")	3,698,606	0	992,824	0
3	Population	S1 OR S2	3,843,712	146.662	1,050,208	28,669
4	Exposure/Comparator	(criteria OR component OR “process based” OR “process-based” OR “process orient*” OR “process-orient*” OR “judgment based” OR behaviour OR “behavioral component” OR “diagnostic assessment” OR screening OR “early identification”)	3,238,958	1.319.341	833,168	155,118
5	Setting/Context	(“clinical physiotherap*” OR “exercise physiolo*” OR “community setting*” OR “community-setting*” OR “community centre” OR “community-centre” OR “community based” OR “community-based” OR home OR “home-based” OR “home based” OR “age* care” OR “aged-care” OR hospital OR clinic OR “general practitioner* clinic” OR “GP clinic” OR “research setting”)	2,325,390	400,004	812,520	82,978
6	Instruments	(“Tinetti Performance Oriented Assessment” OR “The miniBEST” OR “The Functional Movement Screen” OR “The Dynamic Gait Index” OR “The Functional Gait Assessment” OR “Berg Balance” OR “sit-to-stand” OR “chair stand” OR “chair-stand” OR “sit to stand” OR “squat”)	17,579	1,959	8,917	11,022
7	Qualitative Measures	(function* OR “functional measure*” OR “functional movement*” OR movement* OR “independent movement*” OR “independent living movement*” OR master* OR proficiency OR competence OR capacity OR “motor competency” OR “motor skill*” OR “motor skill competency” OR “fundamental movement skill*” OR “foundation* movement skill”)	5,957,328	1,100,793	769,422	233,220
8	Qualitative Measures	(MH "Motor Skills")	27,600	0	14,563	1,362
9	Qualitative Measures	S9 OR S10	5,969,499	1,100,793	775,455	233,523
10	Combined	S3 AND S4 AND S5 AND S6 AND S9	199	18	119	15
11	Limiters	S3 AND S4 AND S5 AND S6 AND S9: Limiters - English Language; Human; Age Related: Aged: 65+ years	170	14	102	13

*Search conducted on the 3^rd^ of June 2025

**Table 5:** Search Stategy for Embase

Search No.	Concept	Search Terms	Embase (n)*
1	Population	(“older adult*” OR elderly OR elder OR “old age*” OR “65 years old”:ab,ti)	4,564,116
2	Population	(**'aged'**/exp)	4,364,417
3	Population	#1 OR #2	4,564,116
4	Exposure/Comparator	(criteria OR component OR “process based” OR “process-based” OR “process orient*” OR “process-orient*” OR “judgment based” OR behaviour OR “behavioral component” OR “diagnostic assessment” OR screening OR “early identification”:ab,ti)	4,227,692
5	Setting/Context	(“clinical physiotherap*” OR “exercise physiolo*” OR “community setting*” OR “community-setting*” OR “community centre” OR “community-centre” OR “community based” OR “community-based” OR home OR “home-based” OR “home based” OR “age* care” OR “aged-care” OR hospital OR clinic OR “general practitioner* clinic” OR “GP clinic” OR “research setting”:ab,ti)	13,008,126
6	Instruments	(“Tinetti Performance Oriented Assessment” OR “The miniBEST” OR “The Functional Movement Screen” OR “The Dynamic Gait Index” OR “The Functional Gait Assessment” OR “Berg Balance” OR “sit-to-stand” OR “chair stand” OR “chair-stand” OR “sit to stand” OR “squat”:ab,ti)	26,018
7	Qualitative Measures	(function* OR “functional measure*” OR “functional movement*” OR movement* OR “independent movement*” OR “independent living movement*” OR master* OR proficiency OR competence OR capacity OR “motor competency” OR “motor skill*” OR “motor skill competency” OR “fundamental movement skill*” OR “foundation* movement skill”:ab,ti)	9,114,735
8	Qualitative Measures	**('motor performance'**/exp)	108,110
9	Qualitative Measures	#9 OR #10	9,135,826
10	Combined	#3 AND #4 AND #5 AND #6 AND #11	736
11	Limiters	#10 AND [embase]/lim NOT ([embase]/lim AND [medline]/lim) AND [aged]/lim AND [english]/lim AND [humans]/lim	230

*Search conducted on the 3^rd^ of June 2025

**Table 6:** Search Strategy for Web of Science (WoS)

Search No.	Concept	Search Terms	WoS (n)*
1	Population	TS=(“older adult*” OR elderly OR elder OR “old age*” OR “65 years old”:ab,ti)	2,729,211
2	Exposure/Comparator	TS=(criteria OR component OR “process based” OR “process-based” OR “process orient*” OR “process-orient*” OR “judgment based” OR behaviour OR “behavioral component” OR “diagnostic assessment” OR screening OR “early identification”:ab,ti)	11,141,601
3	Setting/Context	TS=(“clinical physiotherap*” OR “exercise physiolo*” OR “community setting*” OR “community-setting*” OR “community centre” OR “community-centre” OR “community based” OR “community-based” OR home OR “home-based” OR “home based” OR “age* care” OR “aged-care” OR hospital OR clinic OR “general practitioner* clinic” OR “GP clinic” OR “research setting”:ab,ti)	2,608,114
4	Instruments	TS=(“Tinetti Performance Oriented Assessment” OR “The miniBEST” OR “The Functional Movement Screen” OR “The Dynamic Gait Index” OR “The Functional Gait Assessment” OR “Berg Balance” OR “sit-to-stand” OR “chair stand” OR “chair-stand” OR “sit to stand” OR “squat”:ab,ti)	23,536
5	Qualitative Measures	TS=(function* OR “functional measure*” OR “functional movement*” OR movement* OR “independent movement*” OR “independent living movement*” OR master* OR proficiency OR competence OR capacity OR “motor competency” OR “motor skill*” OR “motor skill competency” OR “fundamental movement skill*” OR “foundation* movement skill”:ab,ti)	12,564,072
6	Combined	#5 AND #4 AND #3 AND #2 AND #1	284
7	Limiters	#6 and English (Languages)	282

*Search conducted on the 3^rd^ of June 2025

**Table 7:** Data extraction form

**Article Details**	
Title	Title of paper
Author	First author of the paper
Year of publication	Year of publication (e.g., 2017)
Country	Name of country where study was conducted
**Article Characteristics**	
Aims/purpose/objectives of the study	State the aim of paper
Study type	Study design
Study Setting	State the setting of the study
Length of Study	State the length of the study (if longitudinal)
**Participant Characteristics**	
Population Description	Outline any relevant co-morbidities
Inclusion Criteria	Describe the inclusion criteria
Exclusion Criteria	Describe the exclusion criteria
Sample Size	Provide the sample size
** Assessment Details**	
Assessment Tool(s) used	Please provide the name(s) of tools used for mobility/functional movement assessment
Type of Task	Describe the task performed
Data Collection Method	Describe the method used to collect the data
**Key Findings/Relevance**	
Summary of Findings	Summarise the findings of the study
Baseline Population Details	Please provide details around age, BMI and sex
**Specific Characteristics of Process-oriented Mobility Tools**	
Tool Name	Name the tool used
Movement Task Assessed	Describe the task assessed
Process-oriented Criteria	Describe the qualitative process-oriented criteria utilised
Scoring Approach	State the scoring approach utilised
Rater Requirements	State whether raters required any training or expertise
Psychometric Properties	State whether any psychometric properties were reported
Feasibility/Acceptability	Describe the feasibility or acceptability of the tool
Technology Used	Describe the technology used in the study (if any)
Strengths of the Tool	Describe the strengths of the tool utilised
Limitations of the Tool	Described the limitations of the tool utilised
Purpose of the Tool	Describe the purpose of the tool

## Abbreviations

BBS: Berg Balance Scale
BESTest: Balance Evaluation Systems Test
DGI: Dynamic Gait Index
FGA: Functional Gait Assessment
FMS: Functional Movement Screen
FRT: Functional Reach Test
ICOPE: Integrated Care for Older People
JBI: Joanna Briggs Institute
mCTSIB: Modified Clinical Test of Sensory Interaction on Balance
Mini-BESTest: Mini-Balance Evaluation Systems Test
OTFACT: Occupational Therapy Functional Assessment Compilation Tool
POMA: Performance-Oriented Mobility Assessment
PRISMA-ScR: Preferred Reporting Items for Systematic Reviews and Meta-Analyses extension for Scoping Reviews
SPPB: Short Physical Performance Battery
TUG: Timed Up and Go
WHO: World Health Organization
