# Supplementary material for: Qualitative assessment of mobility in older adults: a scoping review of process-oriented behavioural criteria
Source: Eur Rev Aging Phys Act. 2026 Apr 9;23:26. doi: 10.1186/s11556-026-00408-y (PMC13343590; doi:10.1186/s11556-026-00408-y)
Supplement: Supplementary file 2 — Supplementary Material 2. [file 11556_2026_408_MOESM2_ESM.docx]

**Supplementary Table S2.** Feasibility, acceptability, efficacy and purpose of process-oriented mobility tools.

| **Author (year)** | **Tool** | **Components of Feasibility/Acceptability** | **Strengths** | **Limitations** | **Purpose** |
| --- | --- | --- | --- | --- | --- |
| Ashburn (2019) (26) | Mini-BEST | Not reported | Not reported | Not reported | Tool used to measure intervention effectiveness |
| Beling (2009) (27) | Posturography | Not reported | Not reported | Not reported | Tool used to measure intervention effectiveness |
| Pavlou (2024) (28) | FGA | FGA assessments take approximately 10-15 minutes, is easy to use for trained clinicians, encompasses a moderate burden on the clinician and participant, and it is generally well-accepted | Not reported | Patients with severe mobility impairments may find some tasks challenging. | Tool used to measure intervention effectiveness |
| Roig-Casasús (2018) (29) | BBS | BBS takes approximately 15-20 minutes to administer, is relatively easy to use for trained clinicians, has a moderate burden on both clinician and participant, and is generally accepted. | Not reported | Not reported | Tool used to measure intervention effectiveness |
| Rosie (2007) (30) | BBS | BBS is quick to administer and is typically simple for a trained clinician to utilise. | Not reported | Not reported | Tool used to measure intervention effectiveness |
| Sihvone (2004) (31) | Posturography & BBS | Both BBS and Posturography were deemed feasible and acceptable | Posturography complete objective measure, no assessor influence or biases | Lack of availability of equipment for Posturography | Tool used to measure intervention effectiveness |
| Sunny (2017) (32) | DGI | Reported to be feasible and acceptable, with a quick administration time (approximately 10-15 minutes), with minimal burden on both clinicians and participants. | Reliable and valid tool for assessing gait in older adults | Not reported | Tool was used for screening; Tool used to measure intervention effectiveness |
| Valiv (2025) (33) | Mini-BEST & FRT | Not reported | Not reported | Not reported | Tool was used for in-depth assessment; Tool used to measure intervention effectiveness |
| Wang (2016) (34) | FMS | Relatively quick to administer and easy to use, can be administer as a remotely monitored home-based assessment. However, no direct measures of acceptability were reported | Relatively quick to administer, can be assessed at home through videoconferencing | Not reported | Tool was used for screening; Tool used to measure intervention effectiveness |
| La Porta (2022) (35) | Mini-BEST & POMA | Not reported | Not reported | Not reported | Tool used to measure intervention effectiveness |
| Millage (2017) (36) | FGA | Not reported | Not reported | Not reported | Tool used to measure intervention effectiveness |
| Tinetti (1997) (37) | OTFACT | Not reported | Not reported | Not reported | Tool was used for in-depth assessment; Feedback gathered from initial assessment was used to personalise intervention; Tool used to measure intervention effectiveness |
| Tisher (2019) (38) | BBS & mCTSIB | Both tools were generally accepted by both patients and clinicians, but mCTSIB requires specific equipment to be administered | Not reported | Not reported | Tool used to measure intervention effectiveness |
| Knobe (2016) (39) | POMA | POMA is a commonly used and simple assessment, although time to administer was not reported it could be inferred that it took a relatively short period of time to administer. No direct patient or clinician acceptability feedback was reported. | Widely used, quick to administer, and requires no equipment | Scoring difficulties alongside poor sensitivity for detecting fallers, with only modest improvement after adjusting cut-offs | Tool was used for screening; Tool was used for in-depth assessment |
| Nikaido (2019) (40) | FGA | Not reported | Useful for predicting falls in patients with neurologic disorders, ceiling effect is less likely to occur, high accuracy for differentiating falls | Difficult to adapt for individuals who require  assistance with walking or standing | Tool was used for screening |
| Panzer (2011) (41) | POMA & Posturography | Not reported | Predictive, objective, multidimensional, and adaptable | Posturography relies on lab-based equipment, and there is a lack of feasibility or acceptability data. | Tool was used for screening |
| Schlenstedt (2016) (42) | Mini-BEST | Mini-BEST was reported to take 10-15 minutes and used readily available equipment, it had a moderate clinician burden as it requires trained examiners and acceptability was not reported. | Multidimensional assessment, standardised administration with trained administrators, short administration time and relatively easy to administer | Does not account for other contributors of falls, is dependent on raters, has tasks which can be challenging for older adults | Tool was used for screening; Tool was used for in-depth assessment |
| Whitney (2013) (43) | POMA & DGI | Both tests require relative minimal equipment and typically feasible to administer to older adults | Ease of administration | Not reported | Tool was used for screening |
| Whitney (2013) (44) | BBS, POMA, DGI, mCTSIB | Not reported | Physical therapists were permitted to choose what they felt was the most appropriate balance and/or gait measurement tool for the patient that they were evaluating | Reliability and validity ratings have had mixed results. Physical therapists permitted to choose what they felt was the most appropriate balance and/or gait measurement tool for the patient | Tool was used for screening; Tool was used for in-depth assessment; Feedback gathered from initial assessment was used to personalise intervention; Tool was used to inform patient/client of their function; Tool used to measure intervention effectiveness |
| Almeida (2014) (45) | DGI | Not reported | Not reported | Not reported | Tool was used for screening |
| Barbieri (2012) (46) | BBS | BBS is a widely used tool in clinical settings, and is relative quick to administer taking approximately 20 minutes to complete | Clinically adopted and accepted, low cost, applicable to multiple populations, no formal certification required, clear protocols and scoring guides available | Ceiling and floor effects, does not assess speed of movements, not disease-specific, requires space and time. | Tool was used to correlate with disease severity |
| Demirbüken (2012) (47) | NeuroCom | Not reported | Capable of detecting centre of gravity sway  during different tasks | Not reported | Tool was used for screening |
| Hayati (2025) (48) | POMA | POMA is typically easy to administer, has a low cost, and is capable of providing valuable insights on balance and gait assessments. However, no direct measures of acceptability were reported. | Reliable, valid, and practical tool for screening frailty, with the added strength of providing cut-off points to guide clinical decision-making. | Scoring requires familiarity with the ordinal scale, and no direct evaluation of acceptability was reported | Tool was used for screening |
| Toledano-Shubi (2025) (49) | Mini-BEST, BBS | Both tests are relatively quick to administer, participant burden can be minimised by optimising efficiency while administering both tools, tasks appearing in both tools were performed only once and scored for each relevant test. | Well-established, validated, and commonly used tools for assessing fall risk and physical function in older adults and could be effectively administered remotely via videoconferencing | Remote testing tended to overestimate walking speed compared to face-to-face assessments.  Difficulties accurately capturing walking speed and gait remotely, possibly due to camera angles, video quality, or timing precision. | Tool was used for screening |

**Abbreviations:** BBS – Berg Balance Scale; DGI – Dynamic Gait Index; FGA – Functional Gait Assessment; FMS – Functional Movement Screen; FRT – Functional Reach Test; Mini-BEST – Mini Balance Evaluation Systems Test; mCTSIB – modified Clinical Test of Sensory Interaction on Balance; OTFACT – Occupational Therapy Functional Assessment Compilation Tool; POMA – Performance Oriented Mobility Assessment.
